# Apigenin Sensitizes Prostate Cancer Cells to Apo2L/TRAIL by Targeting Adenine Nucleotide Translocase-2

**DOI:** 10.1371/journal.pone.0055922

**Published:** 2013-02-19

**Authors:** Masakatsu Oishi, Yosuke Iizumi, Tomoyuki Taniguchi, Wakana Goi, Tsuneharu Miki, Toshiyuki Sakai

**Affiliations:** 1 Department of Molecular-Targeting Cancer Prevention, Graduate School of Medical Science, Kyoto Prefectural University of Medicine, Kawaramachi-Hirokoji, Kamigyo-ku, Kyoto, Japan; 2 Department of Urology, Graduate School of Medical Science, Kyoto Prefectural University of Medicine, Kawaramachi-Hirokoji, Kamigyo-ku, Kyoto, Japan; Bauer Research Foundation, United States of America

## Abstract

Apo2 ligand (Apo2L)/tumor necrosis factor-related apoptosis-inducing ligand (TRAIL) is a promising cancer therapeutic agent. Recombinant human Apo2L/TRAIL has been under clinical trials, whereas various kinds of malignant tumors have resistance to Apo2L/TRAIL. We and others have shown that several anticancer agents and flavonoids overcome resistance to Apo2L/TRAIL by upregulating death receptor 5 (DR5) in malignant tumor cells. However, the mechanisms by which these compounds induce DR5 expression remain unknown. Here we show that the dietary flavonoid apigenin binds and inhibits adenine nucleotide translocase-2 (ANT2), resulting in enhancement of Apo2L/TRAIL-induced apoptosis by upregulation of DR5. Apigenin and genistein, which are major flavonoids, enhanced Apo2L/TRAIL-induced apoptosis in cancer cells. Apigenin induced DR5 expression, but genistein did not. Using our method identifying the direct targets of flavonoids, we compared the binding proteins of apigenin with those of genistein. We discovered that ANT2 was a target of apigenin, but not genistein. Similarly to apigenin, knockdown of ANT2 enhanced Apo2L/TRAIL-induced apoptosis by upregulating DR5 expression at the post-transcriptional level. Moreover, silencing of ANT2 attenuated the enhancement of Apo2L/TRAIL-induced apoptosis by apigenin. These results suggest that apigenin upregulates DR5 and enhances Apo2L/TRAIL-induced apoptosis by binding and inhibiting ANT2. We propose that ANT2 inhibitors may contribute to Apo2L/TRAIL therapy.

## Introduction

Prostate cancer is the second commonly diagnosed cancer and the sixth leading cause of male cancer-related death in the world [1]. Although androgen withdrawal therapy is effective in treating advanced prostate cancer, most patients exhibit resistance to this therapy [2]. It was reported that docetaxel plus prednisone was effective for hormone-refractory prostate cancer [3]. However, the outcome of this treatment is still insufficient [4]. Therefore, new strategies are needed to treat hormone-refractory prostate cancer.

Apo2 ligand (Apo2L)/tumor necrosis factor-related apoptosis-inducing ligand (TRAIL) is a cytokine which belongs to the tumor necrosis factor family. Apo2L/TRAIL binds to death receptor 4 (DR4) and death receptor 5 (DR5) and selectively induces apoptosis in various malignant tumors, but not in normal cells [5], [6]. Recently, several recombinant human Apo2L/TRAIL and agonistic anti-DR5 antibodies were developed, and clinical phase I/II trials have been conducted in patients with many kinds of malignant tumors [7]. However, a number of tumors exhibit resistance to Apo2L/TRAIL and overcoming this resistance is essential for chemotherapy using the Apo2L/TRAIL pathway [8]. We and others have screened dietary compounds sensitizing cancer cells to Apo2L/TRAIL and identified several polyphenols as DR5 inducers [9]–[14]. However, the mechanisms by which these polyphenols upregulate DR5 are unknown.

Adenine nucleotide translocases (ANTs) are important transporters in the inner mitochondrial membrane and play a role in the exchange between ADP and ATP [15]. ANTs have four isoforms in humans and each isoform exhibits a different tissue distribution. ANT2 is overexpressed in many malignant tumor cells and has anti-apoptotic properties [16], [17]. For instance, knockdown of ANT2 has been shown to enhance apoptosis by lonidamine, an antitumor agent targeting mitochondria [17], and induce apoptosis in human breast cancer cells with inhibition of tumor growth *in vivo* [18]. Furthermore, knockdown of ANT2 sensitizes breast cancer cells to Apo2L/TRAIL via upregulation of DR5 [19]. Accordingly, ANT2 is considered to be a promising anticancer target [20], [21].

To elucidate the precise mechanisms through which flavonoids induce DR5 expression, we utilized magnetic FG beads, which recently revealed the target of thalidomide and the mechanism of thalidomide teratogenicity [22], [23]. We identified ANT2 as a binding protein of the dietary flavonoid apigenin which upregulated DR5. As with the treatment of apigenin, knockdown of ANT2 enhanced Apo2L/TRAIL-induced apoptosis by upregulating DR5. Moreover, knockdown of ANT2 attenuated the enhancement of Apo2L/TRAIL-mediated apoptosis by apigenin. In the present study, we first show that ANT2 is a target of apigenin which upregulates DR5 and sensitizes malignant tumor cells to Apo2L/TRAIL.

## Materials and Methods

### Cell Culture

Human prostate cancer cell line DU145 was obtained as a cell line of the NCI-60 from the National Cancer Institute (MD, USA) Developmental Therapeutics Program (NCI DTP). Human prostate cancer cell line LNCaP was obtained from American Type Culture Collection. We have confirmed that these prostate cancer cell lines are negative for mycoplasma using MycoAlert™ Mycoplasma Detection Kit (Lonza, Rockland, ME, USA). DU145 cells were maintained in RPMI 1640 medium supplemented with 10% fetal bovine serum, 2 mmol/L glutamine, 50 units/ml penicillin, and 100 µg/ml streptomycin at 37°C in 5% CO_2_. LNCaP cells were cultured in DMEM medium supplemented with 10% fetal bovine serum, 4 mmol/L glutamine, 50 units/ml penicillin, and 100 µg/ml streptomycin at 37°C in 5% CO_2_.

### Reagents

Apigenin (Wako Pure Chemical Industries, Ltd., Osaka, Japan) and genistein (Cayman Chemical Company, Ann Arbor, MI, USA) were purchased and dissolved in DMSO. Human recombinant Apo2L/TRAIL was purchased from Pepro Tech (London, UK).

### RNAi

The following siRNAs (Invitrogen, Carlsbad, CA, USA) were used: siANT2 #1, 5′-AGAACAUUGGACCAUGCACCCUUGA-3′; siANT2 #2, 5′-UGUAUUGCUUAUCUGCAGUGAUCUG-3′, and Stealth RNAi negative control high GC. Only sense strands are shown. DU145 or LNCaP cells were transfected with siRNAs using Lipofectamine RNAiMAX (Invitrogen).

### Detection of Apoptosis

DU145 or LNCaP cells were treated with various concentrations of each flavonoid for 24 hr or transfected with siRNAs. Human recombinant Apo2L/TRAIL (50 ng/ml) was then added to the cells. After 24 hr, the cells were harvested and suspended in PBS containing 0.1% Triton X-100, 50 µg/ml propidium iodide, and 100 µg/ml RNase A (Sigma, St. Louis, MO, USA). The percentage of hypodiploid DNA (sub-G1) was quantified by FACSCalibur (Becton, Dickinson and Company, Franklin Lakes, NJ, USA). The data were analyzed using Cell Quest software (Becton, Dickinson and Company).

### Western Blot Analysis

DU145 or LNCaP cells were treated with various concentrations of each flavonoid or transfected with siRNAs. The cells were lysed with RIPA buffer (50 mmol/L Tris-HCl [pH 8.0], 150 mmol/L NaCl, 1% NP-40, 0.5% deoxycholic acid, 0.1% SDS, 1 mmol/L DTT, 0.5 mmol/L PMSF) at 4°C for 30 min and were then centrifuged. The supernatants were collected and separated by 10.0% SDS-PAGE and then transferred to Immobilon-P membranes (Millipore, Billerica, MA, USA) using wet method. The membranes were soaked in 5% milk in TBS at 4°C overnight and incubated with primary antibodies at room temperature for 60 min. Primary antibodies were a rabbit polyclonal anti-DR5 antibody (ProSci, Poway, CA, USA) at 1∶500 dilution in 5% milk in TBS and a mouse monoclonal anti-β-actin antibody (Sigma) at 1∶2,000 dilution in 5% milk in TBS. The membranes were then incubated with secondary antibodies conjugated with horseradish peroxidase at 1∶2,000 dilution in TBS-Tween 20 for 60 min at room temperature. Protein bands were visualized on BioMax XAR Film (Carestream Health, Inc., Rochester, NY, USA) using Chemi-Lumi One L (Nacalai Tesque, Kyoto, Japan).

### Quantitative Real-time RT-PCR Analysis

DU145 or LNCaP cells were treated with various concentrations of each flavonoid or transfected with siRNAs. Total RNA was extracted from the cells with Sepasol-RNA I super (Nacalai Tesque). Complementary DNA was synthesized from total RNA using High-Capacity cDNA Reverse Transcription Kits (Applied Biosystems, Melbourne, Australia). Complementary DNA was amplified using TaqMan probes for ANT2, DR5, and GAPDH (Applied Biosystems), and an ABI 7300 Real-time PCR System (Applied Biosystems).

### Preparation of Flavonoid-fixed Beads

Magnetic FG beads with an epoxy linker were purchased from Tamagawa Seiki (Nagano, Japan). Fixation of apigenin and genistein onto the beads was performed as described (Iizumi et al., unpublished data). In brief, the beads were mixed with apigenin in DMF containing potassium carbonate at 37°C for 24 hr as to genistein at 60°C for 24 hr, washed twice with DMF and then twice with deionized water. The resulting beads were stored at 4°C.

### Purification and Identification of Flavonoid-binding Proteins

Flavonoid-fixed beads or empty beads were incubated with DU145 whole cell extracts at 4°C for 4 hr. The binding proteins were eluted with Laemmli dye, subjected to SDS-PAGE, and detected by silver staining. The binding proteins were then subjected to in-gel digestion using Sequencing Grade Modified Trypsin (Promega, Madison, WI, USA). The peptide fragments were analyzed by an Autoflex II mass spectrometer (Burker Daltonics, Billerica, MA, USA).

## Results

### Apigenin Enhances Apo2L/TRAIL-induced Apoptosis via Upregulation of DR5 at the Post-transcriptional Level

**Figure 1:**
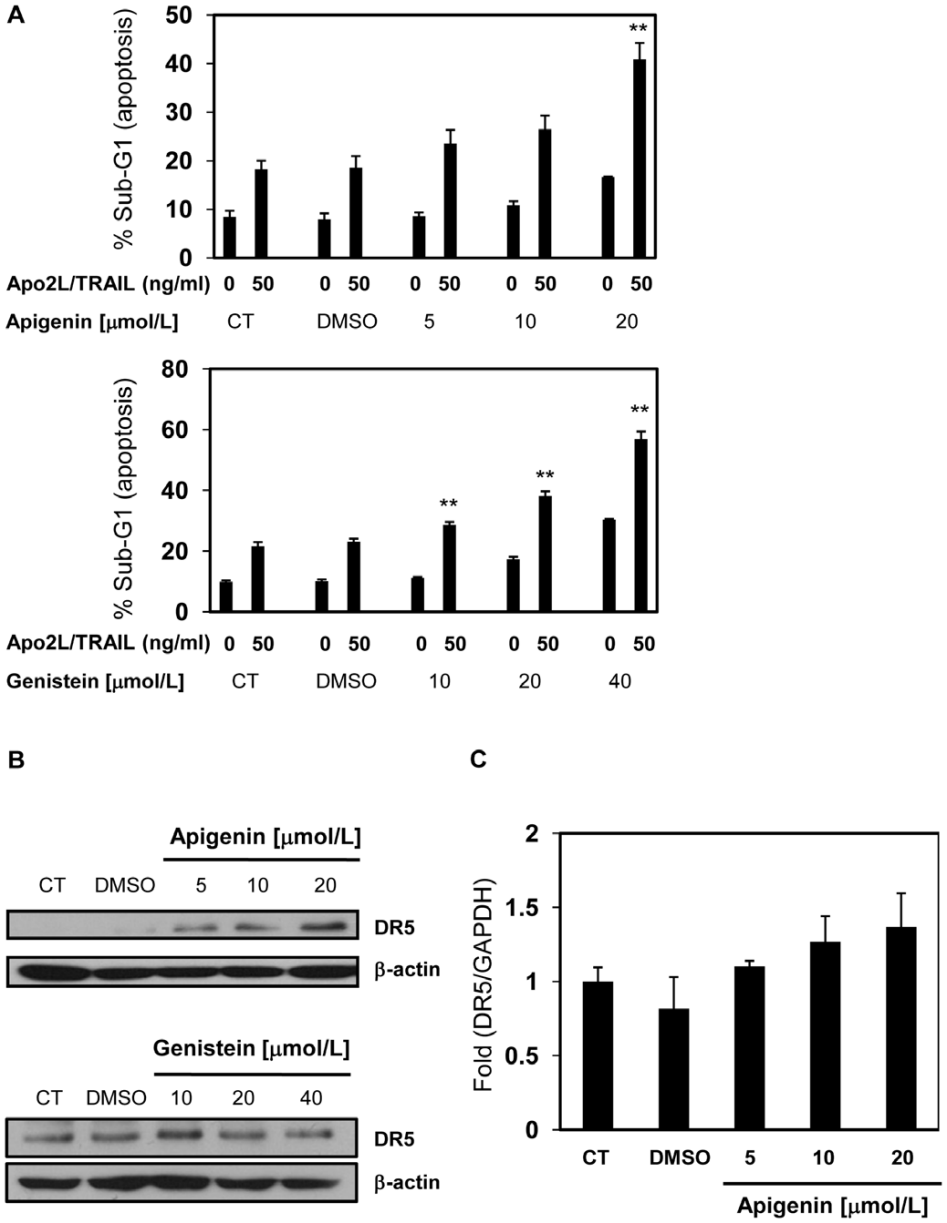
Apigenin enhances Apo2L/TRAIL-induced apoptosis via upregulation of DR5. A, DU145 cells were treated with various concentrations of each flavonoid for 24 hr, followed by treatment with or without 50 ng/ml Apo2L/TRAIL. After 24 hr, the sub-G1 population of the cells was measured using flow cytometry. B, DU145 cells were treated with the indicated concentrations of each flavonoid for 24 hr and harvested. The lysates were analyzed using Western blotting with an anti-DR5 antibody. β-actin was used as a loading control. C, DR5 mRNA was quantified by real-time RT-PCR and normalized by GAPDH. Columns, mean; bars, SD (n = 3). **P<0.01 relative to control.

Among the most common flavonoids, apigenin is known to augment Apo2L/TRAIL-induced apoptosis via upregulation of DR5, while genistein augments Apo2L/TRAIL-induced apoptosis without upregulation of DR5 [9], [24], [25]. In the present study, the combination of these flavonoids and 50 ng/ml Apo2L/TRAIL markedly induced apoptosis, whereas each flavonoid alone did not in human prostate cancer DU145 cells (Fig. 1A, S1, and S2). Apigenin induced DR5 protein expression but genistein did not (Fig. 1B), while apigenin did not upregulate DR5 mRNA (Fig. 1C and S3). These results suggest that apigenin enhances Apo2L/TRAIL-induced apoptosis by post-transcriptionally upregulating DR5.

### ANT2 is Identified as a Binding Protein of Apigenin, but not Genistein

**Figure 2:**
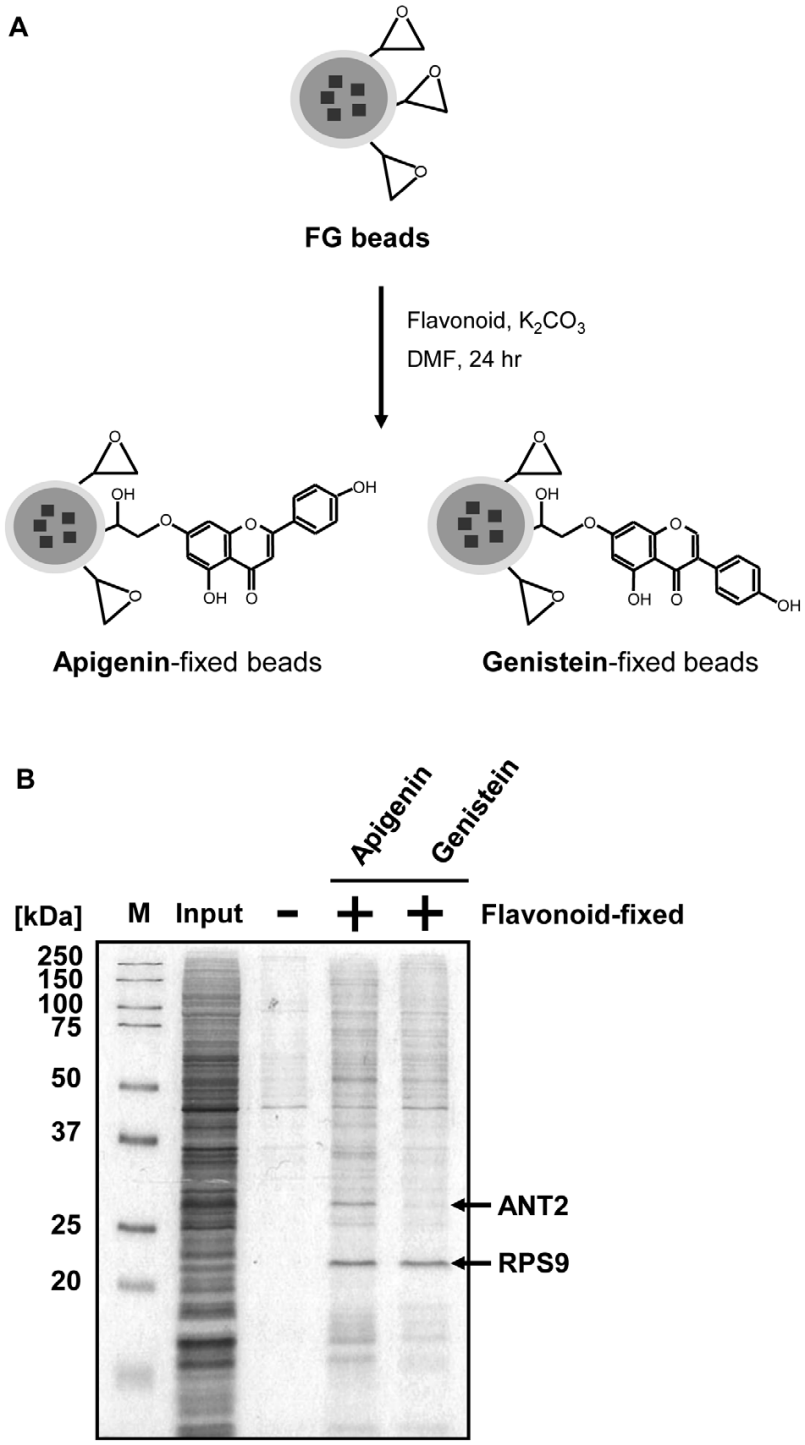
Apigenin, but not genistein, binds to ANT2. A, Fixation of apigenin and genistein onto magnetic FG beads. B, Apigenin- and genistein-binding proteins were purified from whole cell extracts of DU145 cells with each flavonoid-fixed or not (-) FG beads and were analyzed by silver staining. The binding proteins were identified by a mass spectrometer.

To elucidate the mechanism by which apigenin induces DR5 expression, we explored the proteins binding to apigenin, but not genistein, using FG beads with an epoxy linker. Apigenin and genistein were fixed onto these beads (Fig. 2A). The binding proteins of apigenin and genistein were purified from the DU145 whole cell extracts and were identified by MALDI-TOF MS analysis. Adenine nucleotide translocase-2 (ANT2) was identified as a binding protein of apigenin, but not genistein. On the other hand, ribosomal protein S9 (RPS9) was identified as a binding protein of these flavonoids (Fig. 2B). These results indicate that ANT2 is a binding protein of apigenin which induces DR5 expression.

### Knockdown of ANT2 Enhances Apo2L/TRAIL-induced Apoptosis via Upregulation of DR5 at the Post-transcriptional Level

**Figure 3:**
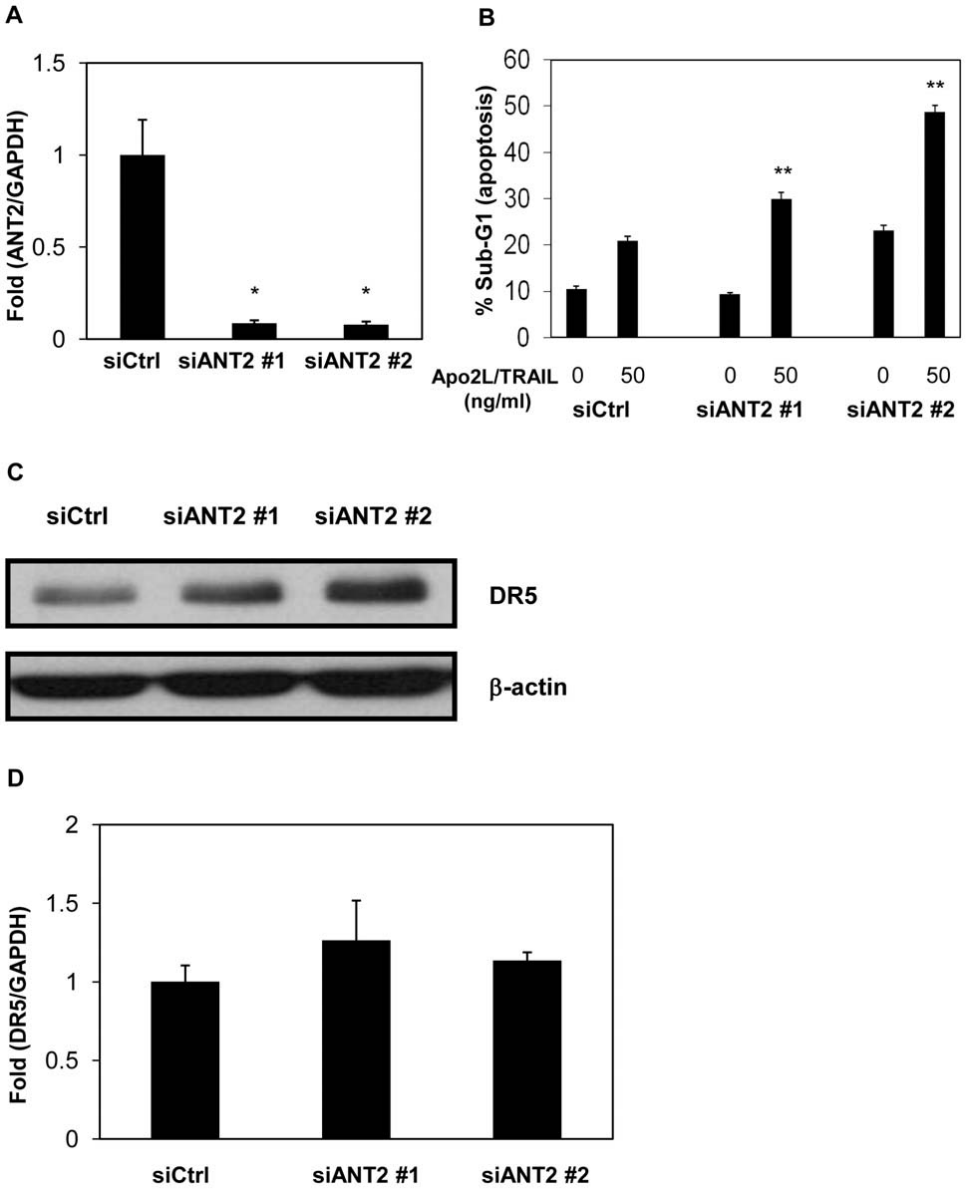
Knockdown of ANT2 enhances Apo2L/TRAIL-induced apoptosis by upregulating DR5 at the post-transcriptional level. DU145 cells were transfected with two different ANT2 siRNAs (siANT2 #1 and #2) or a non-targeting siRNA (siCtrl) and were incubated for 48 hr. A, ANT2 mRNA was quantified by real-time RT-PCR and normalized by GAPDH. B, The cells were treated with or without 50 ng/ml Apo2L/TRAIL. After 24 hr, the sub-G1 population of the cells was measured with flow cytometry. C, Protein levels of DR5 and β-actin were analyzed by Western blotting. D, mRNA levels of DR5 were measured by real-time RT-PCR and normalized by GAPDH. Columns, mean; bars, SD (n = 3). *P<0.05, **P<0.01 relative to control.

It has been reported that knockdown of ANT2 enhances Apo2L/TRAIL-induced apoptosis via upregulation of DR5 in human breast cancer cells [19]. Therefore, we investigated whether knockdown of ANT2 enhanced Apo2L/TRAIL-induced apoptosis in human prostate cancer DU145 cells. ANT2 siRNAs potently repressed the expression of ANT2 mRNA (Fig. 3A and S4). As shown in Fig. 3B and S5, ANT2 siRNAs apparently increased Apo2L/TRAIL-mediated apoptosis. We next examined whether ANT2 knockdown induced DR5 expression. DR5 expression was induced by ANT2 siRNAs in DU145 cells (Fig. 3C), whereas DR5 mRNA was not induced (Fig. 3D and S6). Taken together, these findings suggest that knockdown of ANT2, as well as apigenin, enhances Apo2L/TRAIL-induced apoptosis by post-transcriptionally upregulating DR5.

### Knockdown of ANT2 Attenuates Apo2L/TRAIL-induced Apoptosis Potentiated by Apigenin

**Figure 4:**
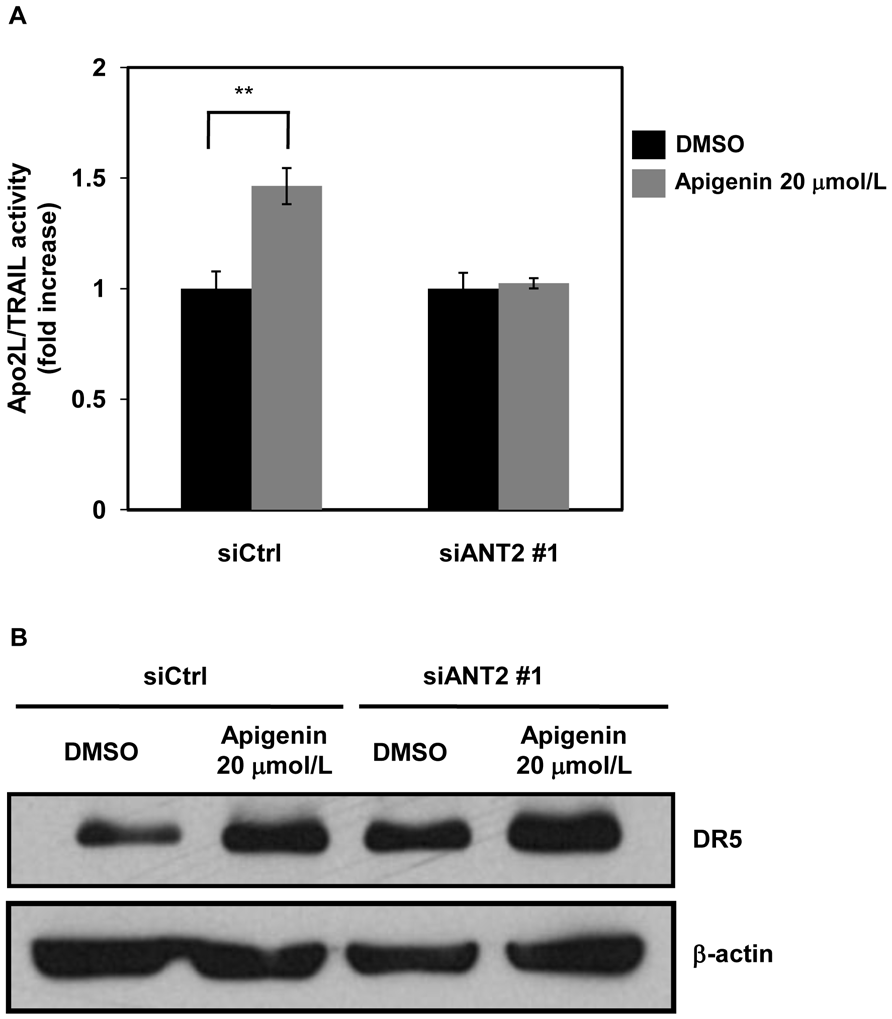
Knockdown of ANT2 suppresses the enhancement of Apo2L/TRAIL-induced apoptosis by apigenin. DU145 cells were transfected with siANT2 #1 or siCtrl. After 48 hr, the cells were incubated with 20 µmol/L apigenin for 24 hr. A, The cells were treated with or without 50 ng/ml Apo2L/TRAIL. After 24 hr, the sub-G1 population of the cells was measured using flow cytometry. The difference in sub-G1 populations with and without Apo2L/TRAIL was defined as Apo2L/TRAIL activity. The Apo2L/TRAIL activity in samples without apigenin was normalized to 1. B, Protein levels of DR5 and β-actin were analyzed by Western blotting. Columns, mean; bars, SD (n = 3). **P<0.01 relative to control.

To elucidate the role of ANT2 in the enhancement of Apo2L/TRAIL sensitivity by apigenin, we examined whether knockdown of ANT2 influenced this enhancement as previously performed as to other target proteins [26], [27]. Apigenin (20 µmol/L) enhanced 50 ng/ml Apo2L/TRAIL-mediated apoptosis in DU145 cells introduced by control siRNA, but not in DU145 cells introduced by ANT2 siRNA (Fig. 4A and S7). Silencing of ANT2 lowered the enhancement of DR5 expression by 20 µmol/L apigenin (Fig. 4B). These results indicate that ANT2 inhibition is required for apigenin to enhance DR5 expression and Apo2L/TRAIL-induced apoptosis.

**Figure 5:**
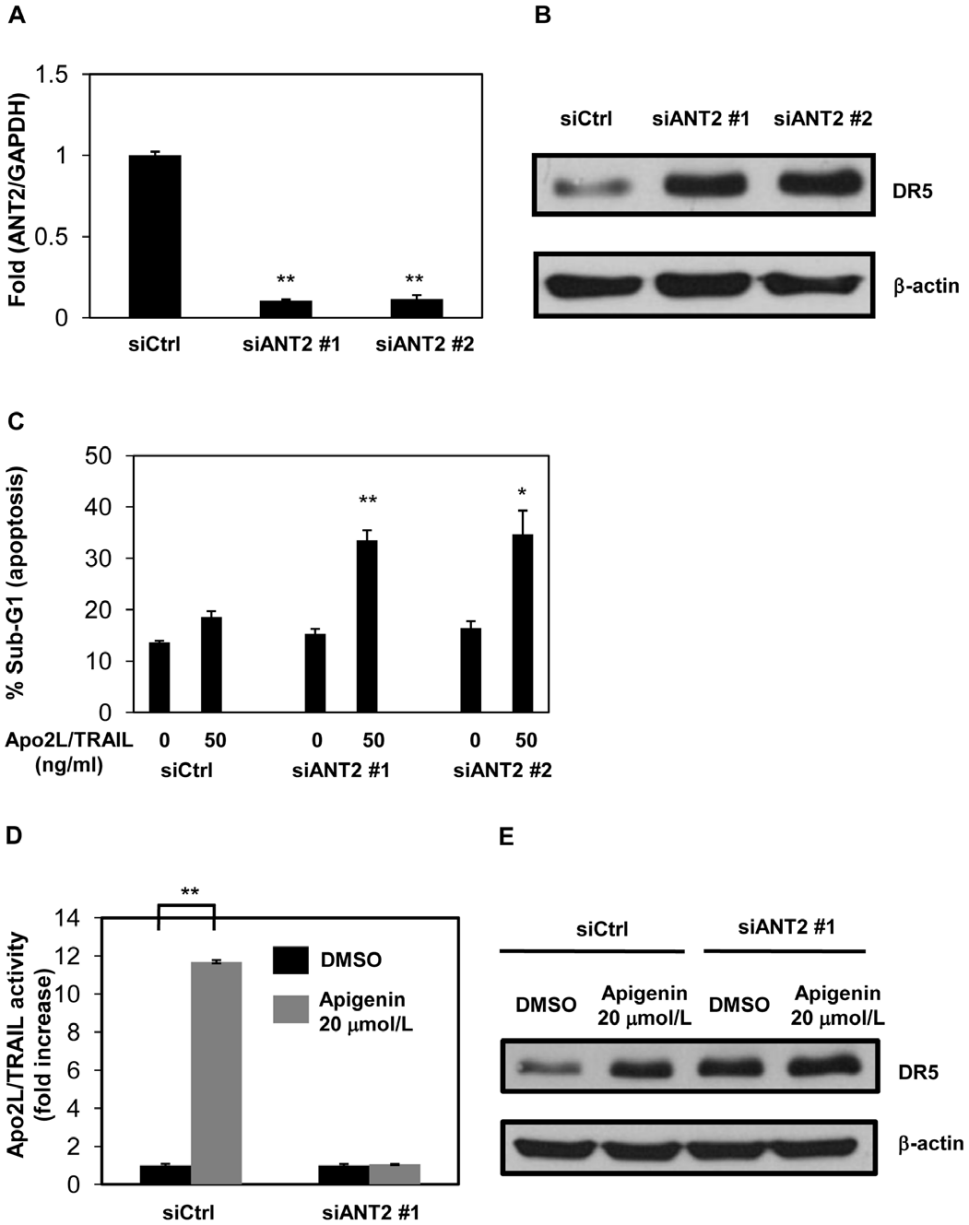
Apigenin enhances Apo2L/TRAIL-induced apoptosis by inhibiting ANT2 in LNCaP cells. LNCaP cells were transfected with siANT2 or siCtrl and were incubated for 48 hr. A, ANT2 mRNA was quantified by real-time RT-PCR and normalized by GAPDH. B, Protein levels of DR5 and β-actin were analyzed by Western blotting. C, The cells were incubated with or without 50 ng/ml Apo2L/TRAIL. After 24 hr, the sub-G1 population of the cells was measured with flow cytometry. D, The cells were incubated with 20 µmol/L apigenin for 24 hr, followed by treatment with or without 50 ng/ml Apo2L/TRAIL. The Apo2L/TRAIL activity in samples without apigenin was normalized to 1. E, The cells were incubated with 20 µmol/L apigenin for 24 hr. Protein levels of DR5 and β-actin were analyzed by Western blotting. Columns, mean; bars, SD (n = 3). *P<0.05, **P<0.01 relative to control.

We next investigated the role of ANT2 in another cancer cell line, hormone-dependent human prostate cancer LNCaP. ANT2 mRNA was effectively silenced by ANT2 siRNAs (Fig. 5A and S8), and knockdown of ANT2 also induced DR5 expression (Fig. 5B) and enhanced 50 ng/ml Apo2L/TRAIL-induced apoptosis (Fig. 5C and S9). In addition, knockdown of ANT2 attenuated the enhancement of Apo2L/TRAIL sensitivity and DR5 expression by 20 µmol/L apigenin (Fig. 5D, E, and S10).

## Discussion

**Figure 6:**
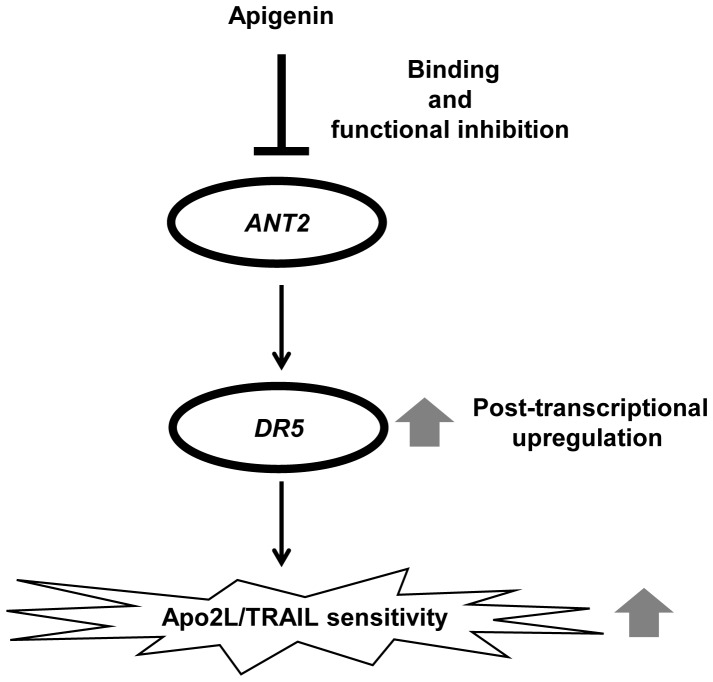
Schematic model of the enhancement of Apo2L/TRAIL sensitivity by apigenin. Apigenin binds and inhibits ANT2. Inhibition of ANT2 augments Apo2L/TRAIL sensitivity via upregulation of DR5.

In the present study, we identified ANT2 as a binding protein of apigenin which upregulated DR5 using magnetic FG beads (Fig. 2). Knockdown of ANT2 enhanced Apo2L/TRAIL-induced apoptosis by upregulating DR5 (Fig. 3), similar to treatment with apigenin (Fig. 1). Moreover, knockdown of ANT2 attenuated the enhancement of DR5 expression and Apo2L/TRAIL-induced apoptosis by apigenin (Fig. 4 and 5). These results suggest that apigenin binds and inhibits ANT2, which induces DR5 expression and potentiates Apo2L/TRAIL sensitivity (Fig. 6). The present study also suggests that apigenin post-transcriptionally induces DR5 by inhibiting ANT2 (Fig. 1B and C). We suppose that several agents, which have been reported to upregulate DR5 at the transcriptional level [10]–[13], might also induce DR5 expression at the post-transcriptional level by inhibiting ANT2.

It has been reported that knockdown of ANT2 elevates ROS levels [17] and activates JNK and p53 [19], consistent with that apigenin also increases ROS levels [25], [28] and activates JNK [29] and p53 [30]. In addition, the present study showed that apigenin bound to ANT2 and upregulated DR5 similar to knockdown of ANT2. These results strongly suggest that apigenin functionally inhibits ANT2.

Several polyphenols, such as apigenin, have been reported to enhance Apo2L/TRAIL sensitivity via various pathways [25], [31]. However, the detailed mechanisms through which each polyphenol increases Apo2L/TRAIL sensitivity remain unknown. Our method identifying the targets of polyphenols may be useful for elucidating these mechanisms.

We elucidated the mechanism through which apigenin upregulated DR5 by comparing the binding proteins of apigenin that upregulated DR5 and genistein that did not (Fig. 2). The strategy that compares the direct binding proteins of various agents is beneficial. For instance, it has been clarified that ligands of VDAC proteins selectively induce non-apoptotic cell death in tumor cells harbouring mutations in the oncogenes *HRAS*, *KRAS*, or *BRAF* by comparing the binding proteins of erastin A6 and erastin B2, an erastin analogue that lacks its activity [32]. Comparison of the binding proteins of different agents may reveal the proteins that cause the main or adverse effects of these agents. Moreover, pharmacological control of these binding proteins may develop current chemotherapy.

To date, several agonistic anti-DR5 antibodies and human recombinant Apo2L/TRAIL have been developed and are under clinical trials, whereas some cancers have exhibited resistance to these agents [7]. In the present study, knockdown of ANT2 sensitized cancer cells to Apo2L/TRAIL by upregulating DR5. This study also suggests that apigenin may be an ANT2 inhibitor. Accordingly, apigenin and novel ANT2 inhibitors may enhance the effects of these agents by overcoming resistance to Apo2L/TRAIL.

## Supporting Information

Figure S1 **The histograms of** **Figure 1A** **as to apigenin.**
(TIF)

Figure S2 **The histograms of** **Figure 1A** **as to genistein.**
(TIF)

Figure S3 **The amplification curves of** **Figure 1C** **.**
(TIF)

Figure S4 **The amplification curves of** **Figure 3A** **.**
(TIF)

Figure S5 **The histograms of** **Figure 3B** **.**
(TIF)

Figure S6 **The amplification curves of** **Figure 3D** **.**
(TIF)

Figure S7 **The histograms of** **Figure 4A** **.**
(TIF)

Figure S8 **The amplification curves of** **Figure 5A** **.**
(TIF)

Figure S9 **The histograms of** **Figure 5C** **.**
(TIF)

Figure S10 **The histograms of** **Figure 5D** **.**
(TIF)
