# Supplementary figures and images for: Apigenin Sensitizes Prostate Cancer Cells to Apo2L/TRAIL by Targeting Adenine Nucleotide Translocase-2
Source: PLoS One. 2013 Feb 19;8(2):e55922. doi: 10.1371/journal.pone.0055922 (PMC3576345; doi:10.1371/journal.pone.0055922)

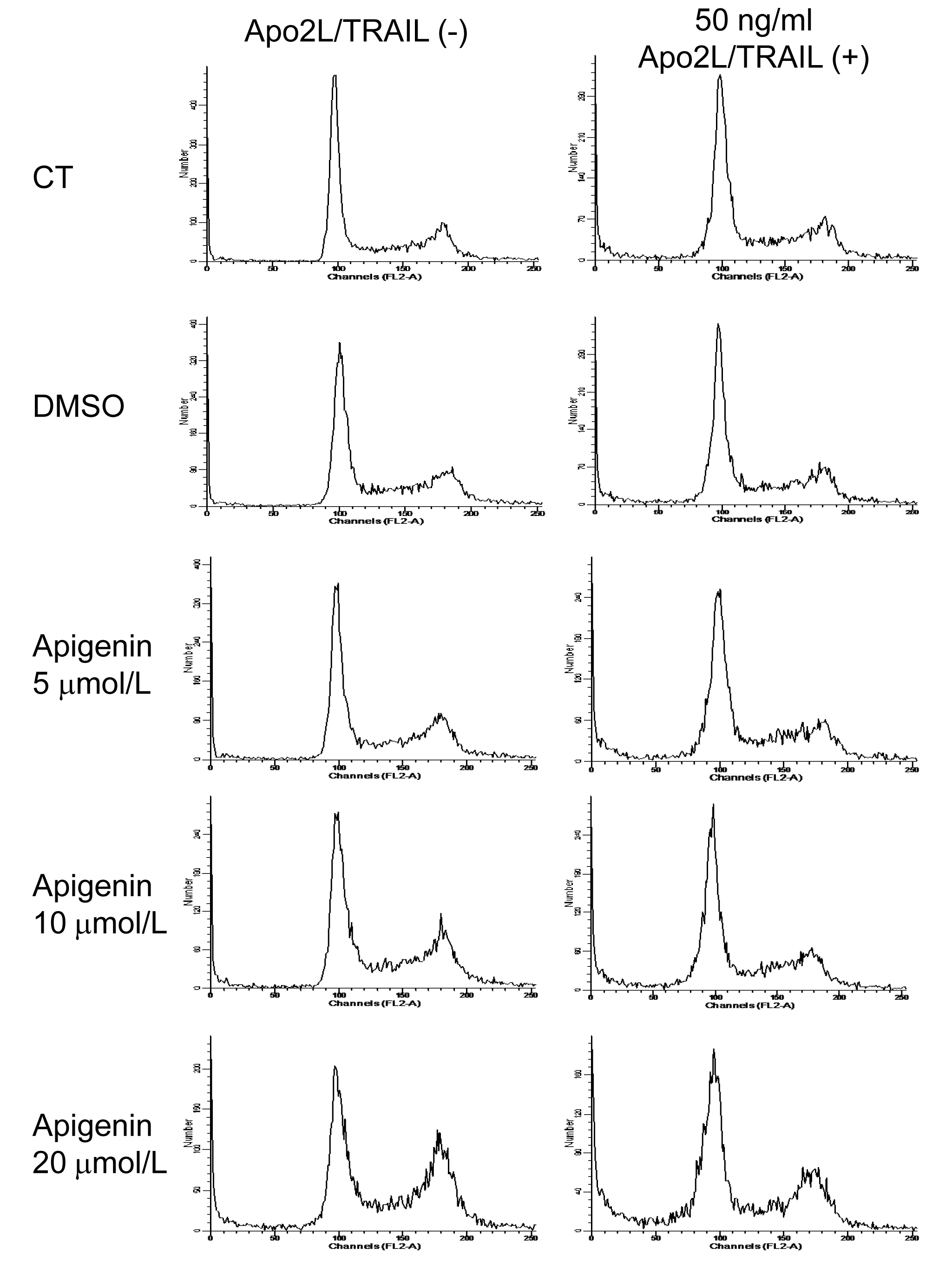

Supplement: Figure S1 — The histograms of Figure 1A as to apigenin. (TIF) [file pone.0055922.s001.tif]

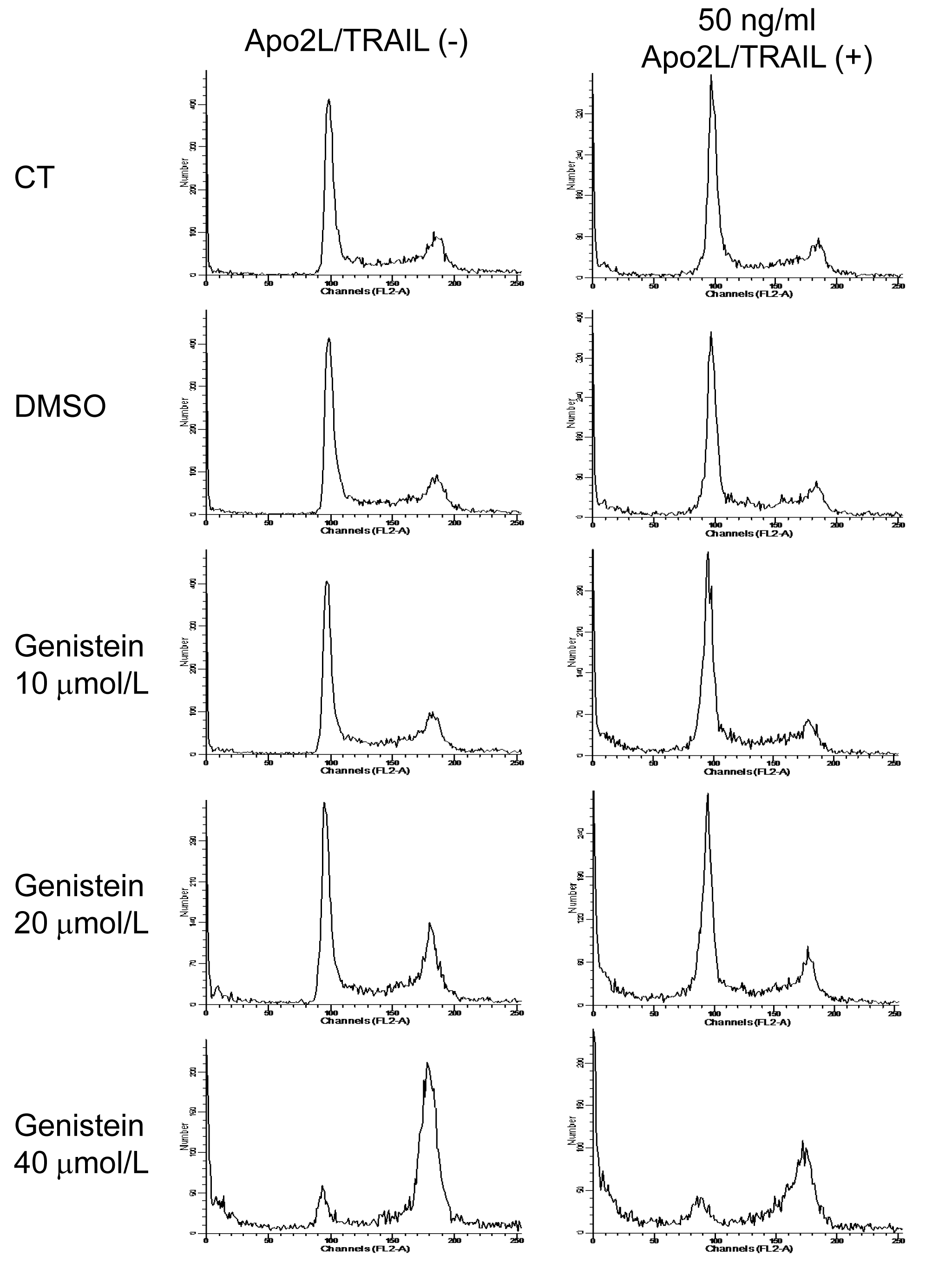

Supplement: Figure S2 — The histograms of Figure 1A as to genistein. (TIF) [file pone.0055922.s002.tif]

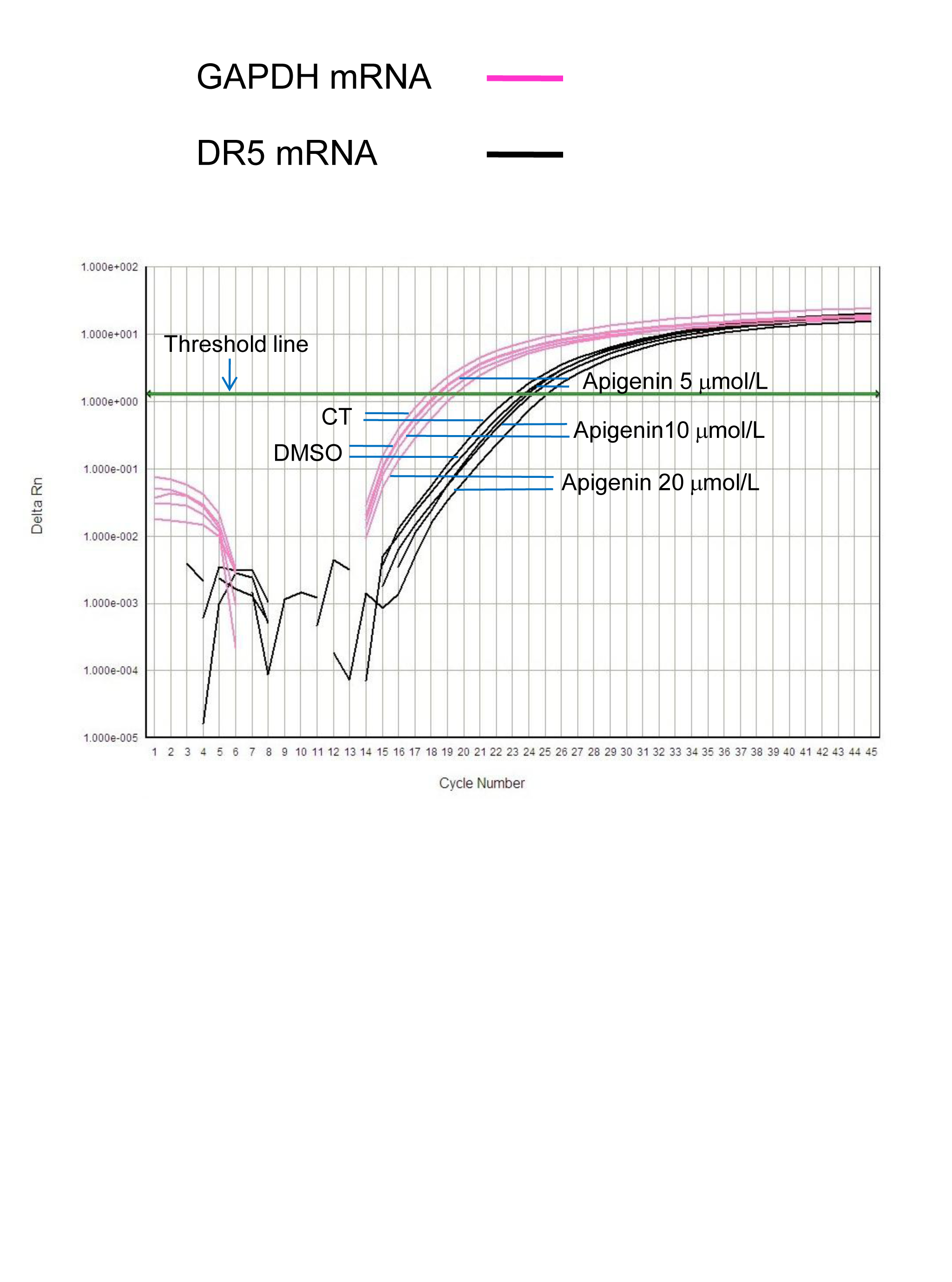

Supplement: Figure S3 — The amplification curves of Figure 1C . (TIF) [file pone.0055922.s003.tif]

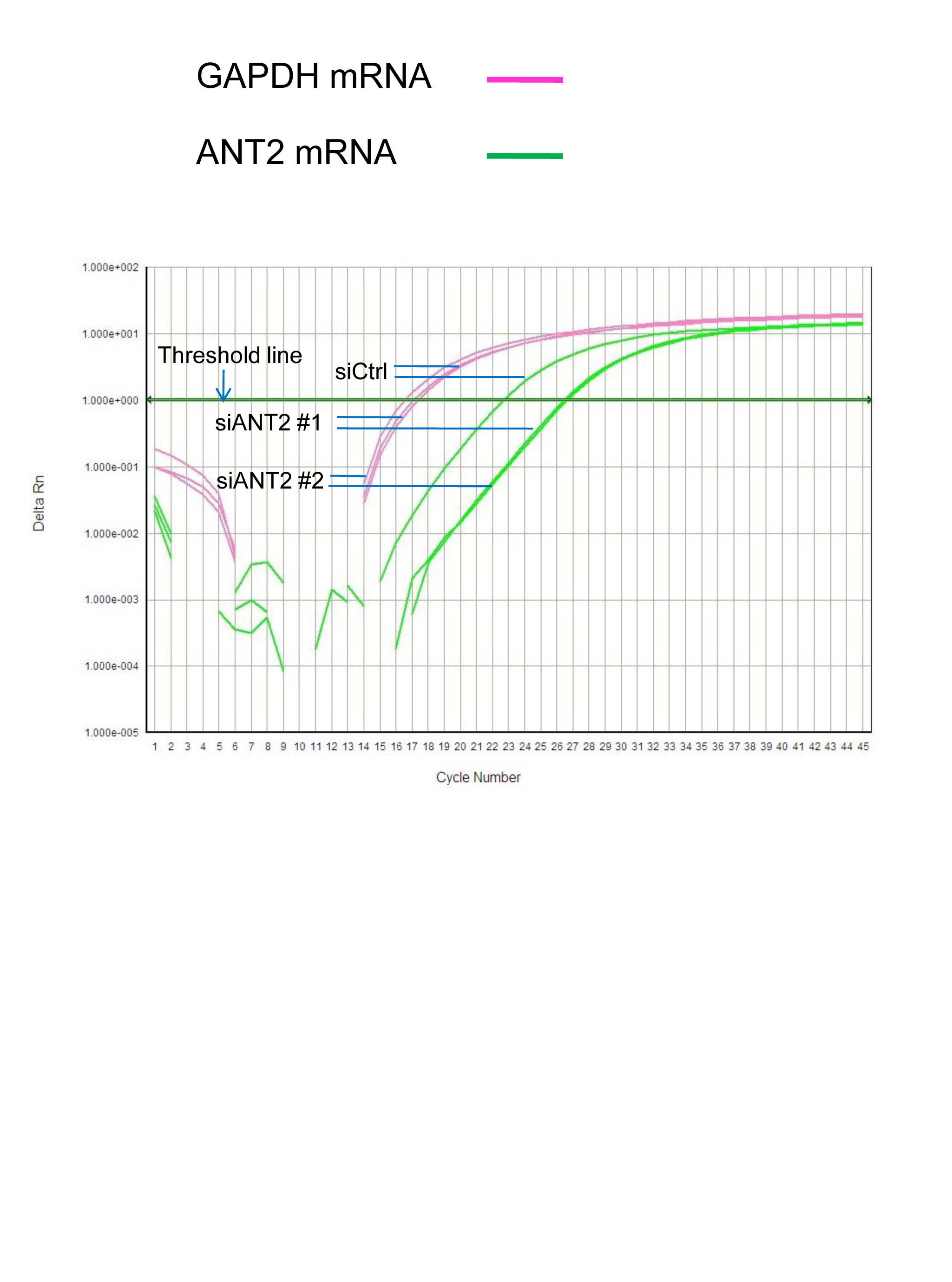

Supplement: Figure S4 — The amplification curves of Figure 3A . (TIF) [file pone.0055922.s004.tif]

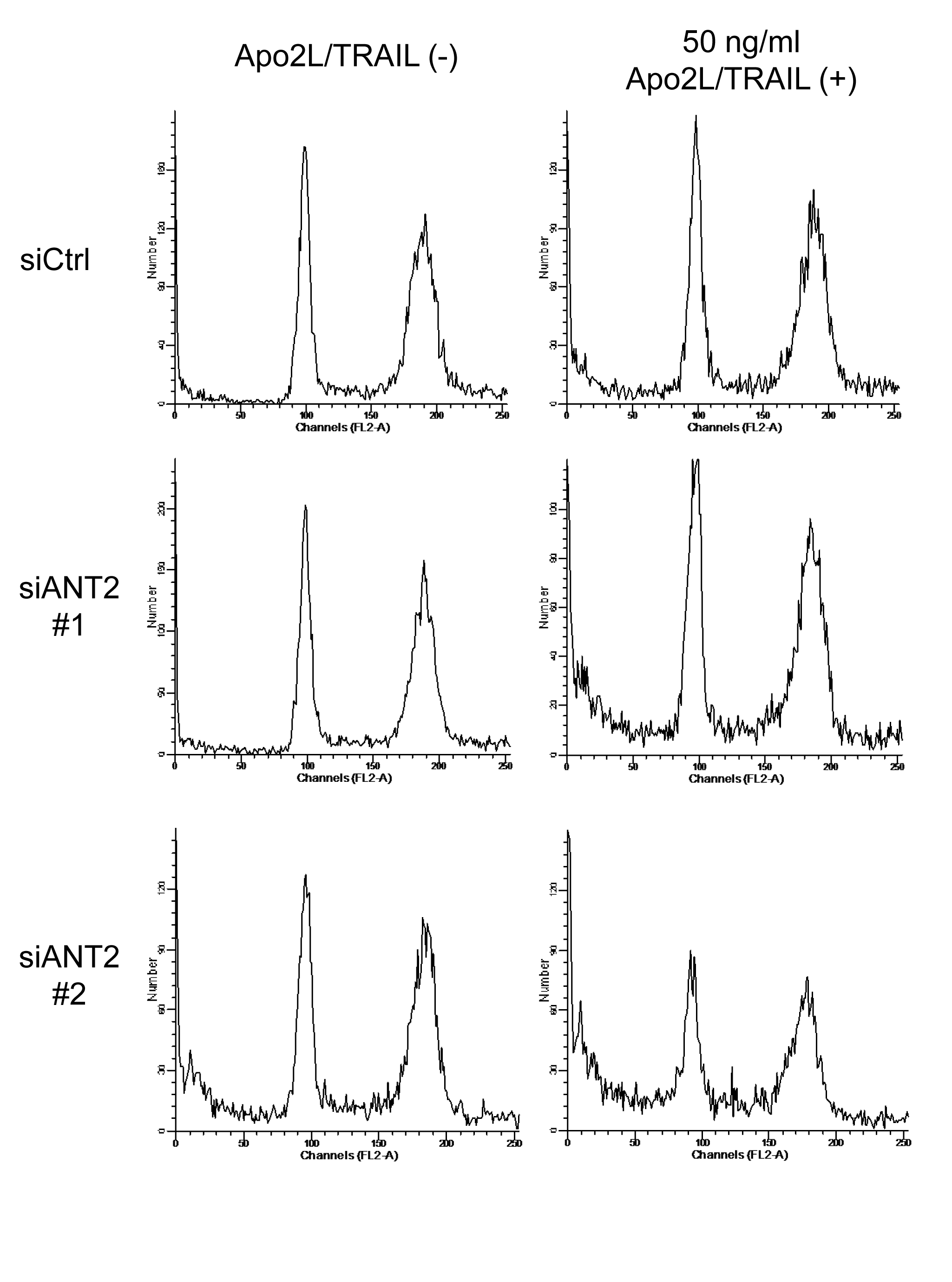

Supplement: Figure S5 — The histograms of Figure 3B . (TIF) [file pone.0055922.s005.tif]

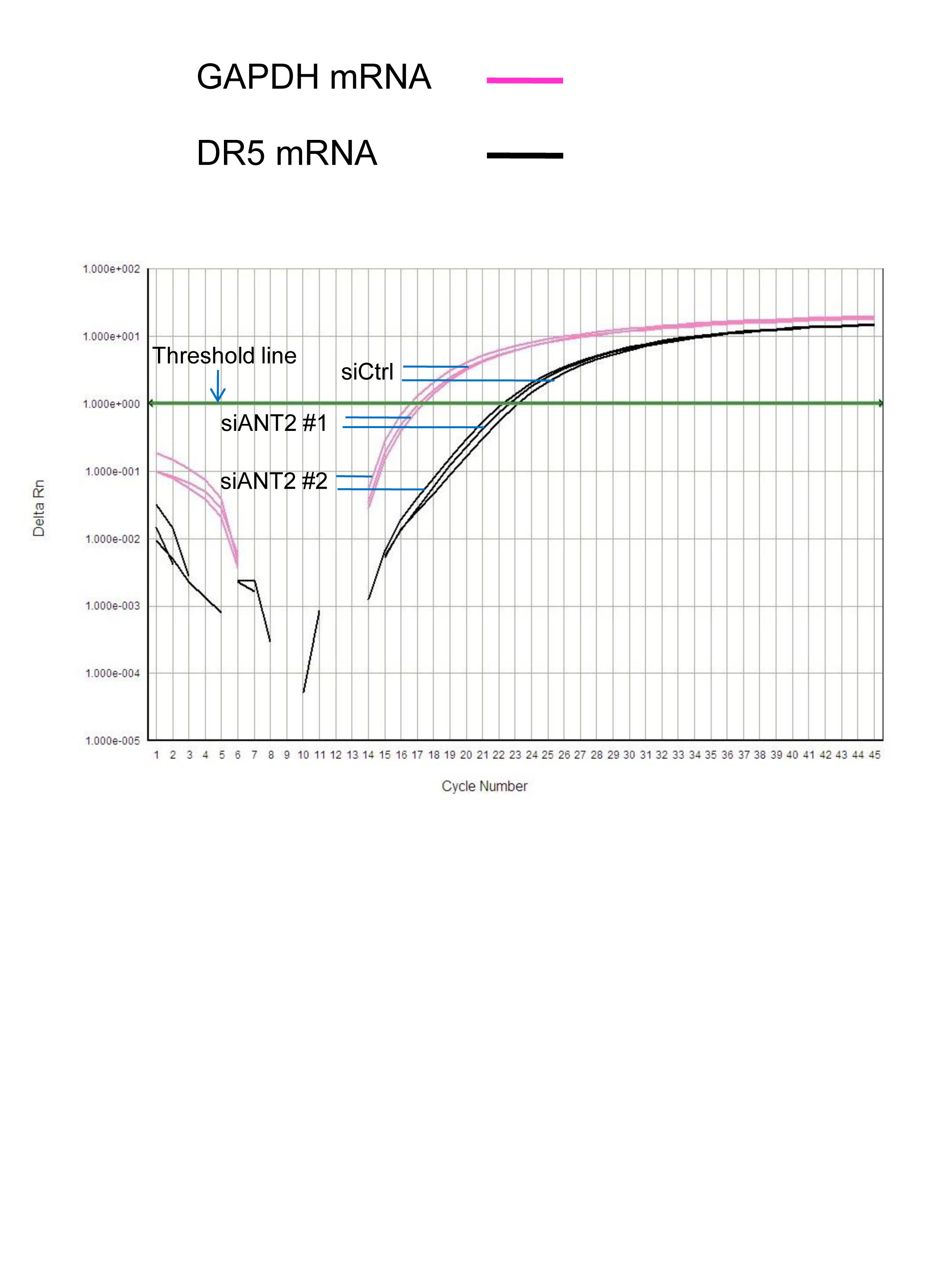

Supplement: Figure S6 — The amplification curves of Figure 3D . (TIF) [file pone.0055922.s006.tif]

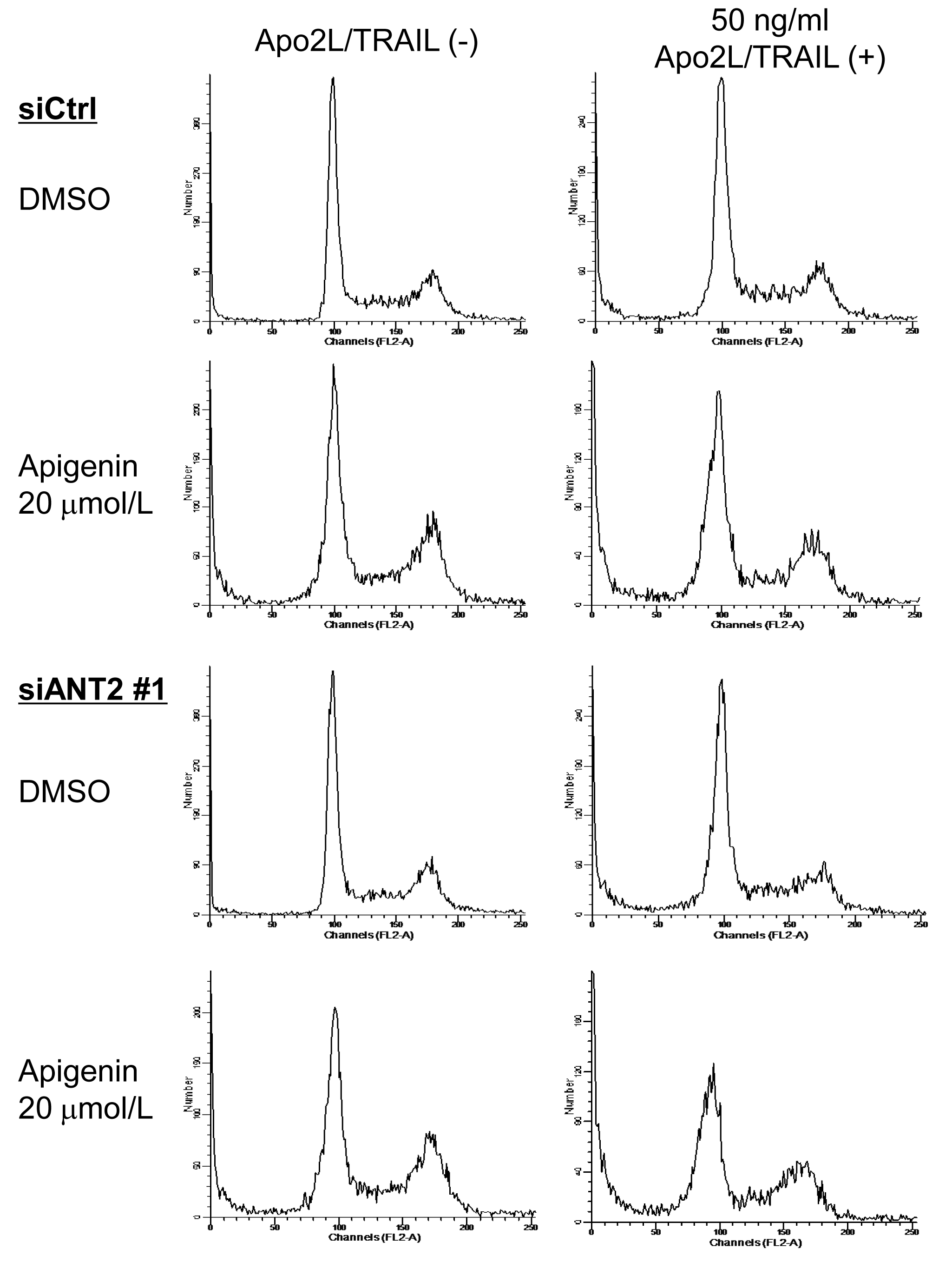

Supplement: Figure S7 — The histograms of Figure 4A . (TIF) [file pone.0055922.s007.tif]

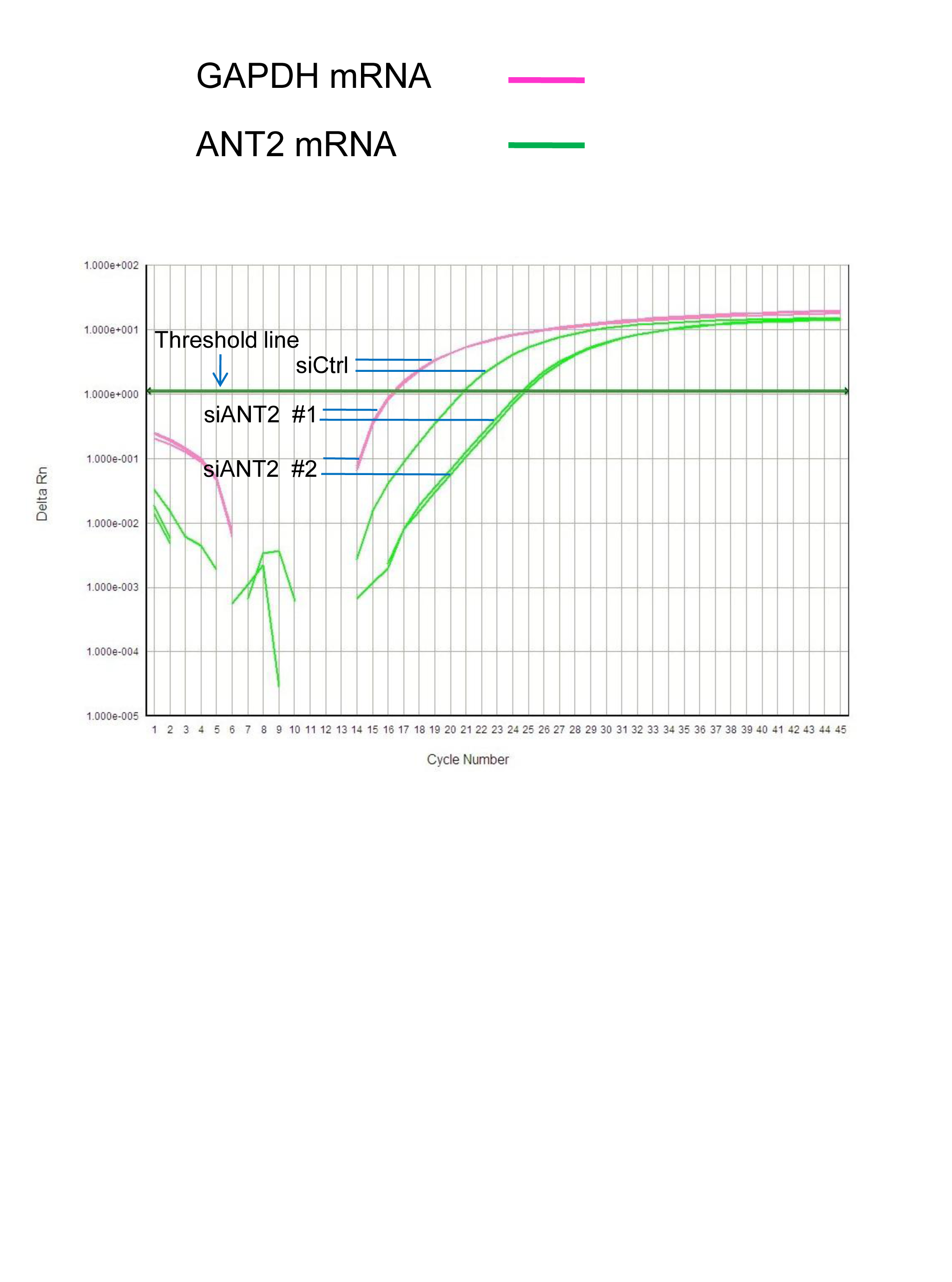

Supplement: Figure S8 — The amplification curves of Figure 5A . (TIF) [file pone.0055922.s008.tif]

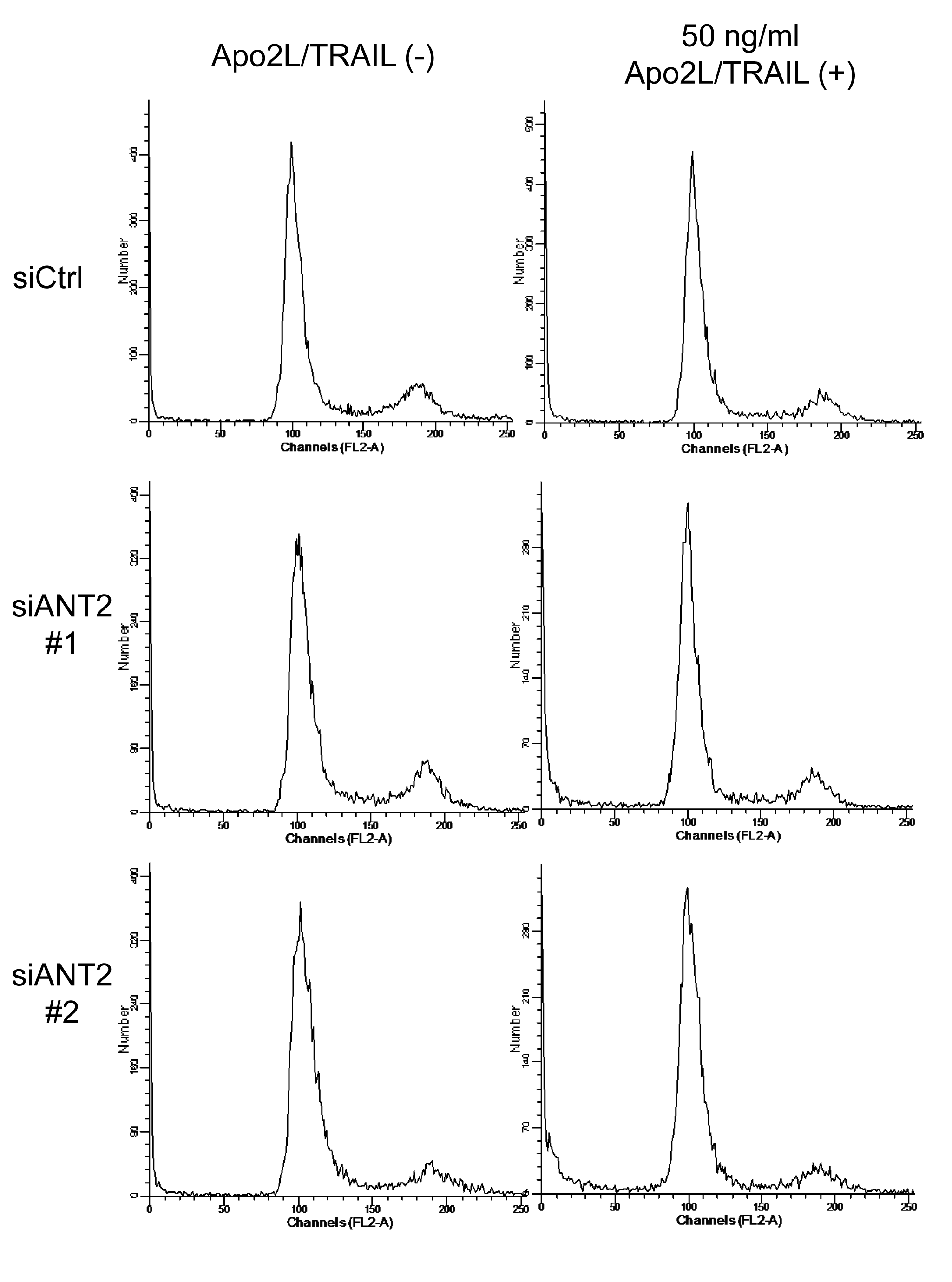

Supplement: Figure S9 — The histograms of Figure 5C . (TIF) [file pone.0055922.s009.tif]

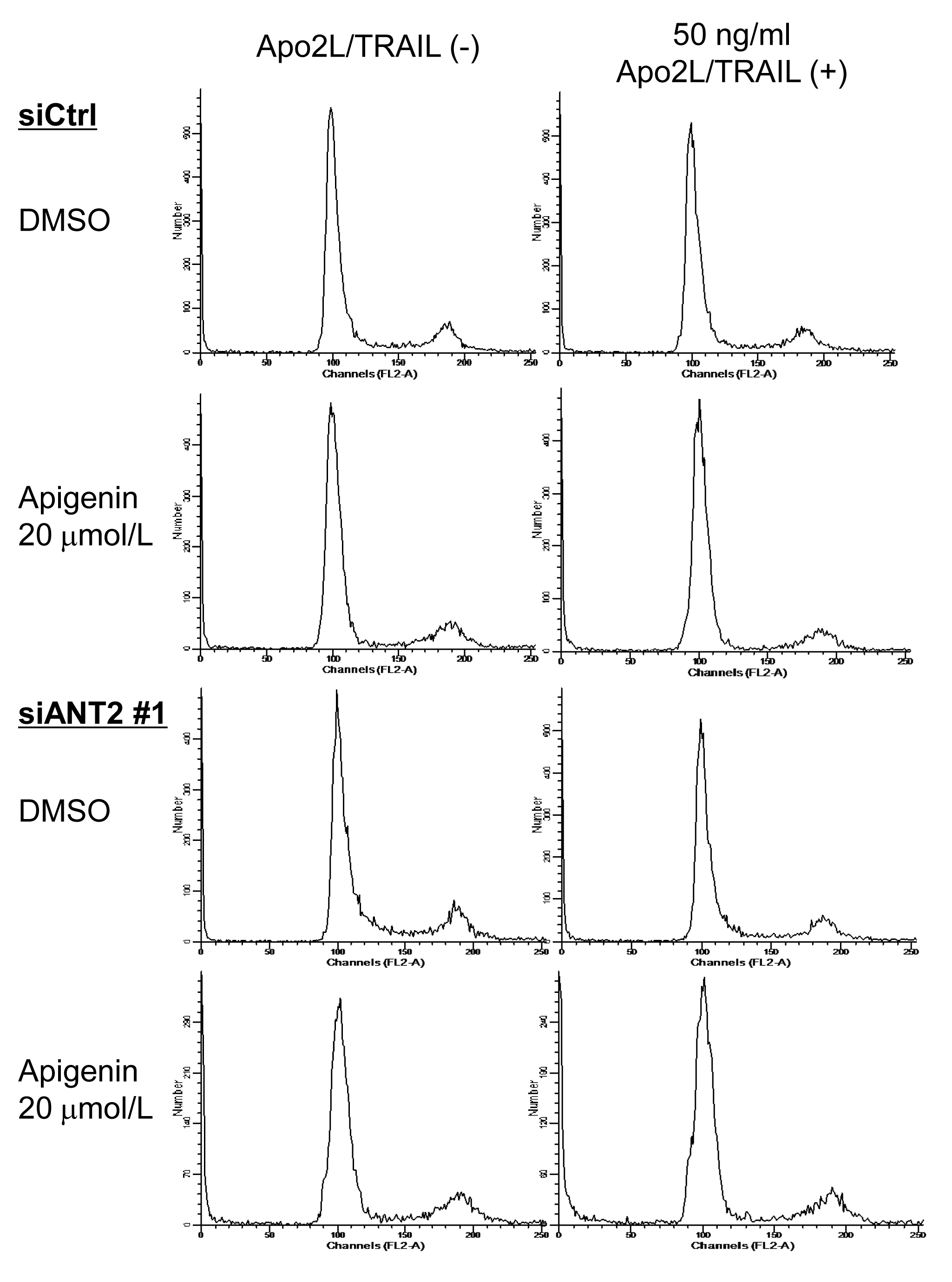

Supplement: Figure S10 — The histograms of Figure 5D . (TIF) [file pone.0055922.s010.tif]
